# Telomere-driven dysfunctional changes in gynecological cancers: mechanistic insights, biomarker potential, and therapeutic targeting

**DOI:** 10.3389/fcell.2026.1797677

**Published:** 2026-06-03

**Authors:** Olaolu Ayoade Afolabi, Timileyin Abdul-Mumeen Alade, Kehinde Samuel Olaniyi, Mary Oluwalewa Adeyemi, Seun Oladepo, Joshua Oluwasola Gbadero, Marvelous Dasola Oyedokun, Sola Babatunde Olayemi, Blessing Monica Akindele, Ayoola Abimbola Oladipo, Cecelia Adedeji Adegbola, Onome Bright Oghenetega, Sidiquot Yetunde Akinsanya, John Sotunsa, Olajumoke Deborah Ogunleye, Abiodun Shukurat Lasisi-Sholola, Victor Pelumi Owolabi, Oluwatobi Adewale, James Adewale, Deborah Adeola Oke, Habeeb Adelani Abdur-Rahman, Precious Adeoye Oyedokun

**Affiliations:** 1 Department of Obstetrics and Gynecology, Bowen University, Iwo, Osun, Nigeria; 2 Reproductive Biology and Toxicology Research Laboratory, Oasis of Grace Hospital, Osogbo, Osun, Nigeria; 3 Department of Physiology, Ladoke Akintola University of Technology, Ogbomoso, Oyo, Nigeria; 4 Axon Plus Research Consortium, Ogbomoso, Oyo, Nigeria; 5 Department of Physiology, Afe Babalola University, Ado-Ekiti, Ekiti, Nigeria; 6 Department of Medical Laboratory Science, Ladoke Akintola University of Technology, Ogbomoso, Oyo, Nigeria; 7 Department of Nursing Science, Ladoke Akintola University of Technology, Ogbomoso, Oyo, Nigeria; 8 Department of Physiology, Adeleke University, Ede, Osun, Nigeria; 9 Department of Medical Laboratory Science, Fountain University, Osogbo, Oyo, Nigeria; 10 Department of Obstetrics and Gynecology, Benjamin Carson (SNR) College of Health and Medical Sciences, Babcock University, Ilisha Remo, Ogun, Nigeria; 11 Gullas College of Medicine, University of Visayas, Cebu, Philippines; 12 Department of Biochemistry, Ladoke Akintola University of Technology, Ogbomoso, Oyo, Nigeria; 13 Center of Excellence for Aging and Brain Repair, University of South Florida, Tampa, FL, United States; 14 Department of Physiology, Redeemers University, Ede, Osun, Nigeria; 15 Department of Anatomy, Adeleke University, Ede, Osun, Nigeria

**Keywords:** chromosomal integrity, genomic instability, gynecological cancers, telomerase, telomere

## Abstract

Telomeres are major determinants of chromosome integrity, and when poorly regulated, they have been shown to contribute critically to malignant transformation. However, in gynaecological malignancies, telomeres are interpreted inconsistently and have yet to be smoothly incorporated into diverse disease types. The review incorporates the existing evidence of the relationship between telomere dysfunction and the evolution of cervical, ovarian, endometrial, vulvar, and vaginal cancer. The review discusses the joint causes of telomere erosion, defective capping, defective integrity, and dysregulated telomerase activity in gynaecological cancer development, and the ensuing genomic instability, disruption in tissue homeostasis, inflammation, immune evasion, clonal evolution, and changes in individual organ physiology. According to this integrative framework, there are common and cancer-specific processes, new biomarkers for telomere maintenance, and therapeutic prospects focused on telomere maintenance pathways. The development of standardized, longitudinal methodologies will be key to translating telomere biology into clinically actionable approaches in gynaecological oncology.

## 1 Introduction

Gynaecological cancers, including ovarian, endometrial, cervical, vulvar, and vaginal malignancies, represent a major component of the global cancer burden among women. Collectively, they account for approximately 1,473,427 new cases and 680,372 deaths globally in 2022 (Zhu et al., 2024). Cervical cancer remains one of the most commonly diagnosed gynaecological malignancies globally, with approximately 662,301 new cervical cancer cases and 348,874 cervical cancer deaths annually (Son et al., 2025). Cervical cancer is a leading cause of cancer-related mortality among women in low- and middle-income countries (Son et al., 2025). In contrast, endometrial cancer is the most frequently diagnosed gynaecological cancer in high-income regions, with over 400,000 new cases each year, and its global incidence continues to rise in parallel with increasing obesity, metabolic disease, and population ageing (Crosbie et al., 2022). In 2018, 184,799 deaths occurred due to ovarian cancer, accounting for 4.4% of the entire cancer-related mortality among women (Momenimovahed et al., 2019). Vulvar and vaginal cancers are relatively rare tumors worldwide, with 44,000 and 17,500 new cases estimated in 2018 respectively, but they contribute disproportionately to morbidity in older women and in populations with persistent human papillomavirus (HPV) infection (Nithyanandhan et al., 2025).

Despite progress in prevention, screening and treatment, survival outcomes remain poor in many gynecological cancers (Ruzindana and Anorlu, 2025). Ovarian cancer still has a poor rate of long-term survival, as most of the disease is diagnosed in its advanced stages (Ali et al., 2023). Cervical cancer, though largely preventable through vaccination and screening, continues to result in a significant number of deaths annually, indicating persistent global inequities in access to healthcare (Wu et al., 2025). Even in endometrial cancer, which is usually diagnosed early, aggressive subtypes and recurrent disease are significant clinical problems. These epidemiological realities justify an urgent need for improved molecular biomarkers, prevention strategies, and novel therapeutic approaches (Renaud et al., 2025).

Genomic instability is a key characteristic that is fundamental to the onset and progression of gynaecological cancers. It drives the accumulation of genetic mutations, the structural chromosomal alterations, and aneuploidy, which contribute to the heterogeneity of tumors, diseases, and resistance to therapies (Ferguson et al., 2015). Among the molecular structures important for maintaining genomic integrity are telomeres. Normal telomere structure protects the chromosome ends from being recognized as DNA damage, and different mechanisms can cause progressive telomere shortening, loss of shelterin-mediated protection, or aberrant telomere maintenance. Telomeric damage arises primarily from oxidative stress, while telomere shortening results from the end-replication problem (ERP) during normal DNA replication. These processes cumulatively trigger DNA damage responses and chromosomal instability (Rembiałkowska et al., 2025). Telomere dysfunction has been implicated at the earliest stages of gyneacological carcinogenesis and is involved in malignant transformation, tumor progression, and resistance to treatment (Nassour and Karlseder, 2025).

Telomere biology has specific relevance to gynaecological oncology because of its close association with female reproductive physiology. The ovary, endometrium, and cervix undergo cycles of continuous proliferation and regeneration, which rely on tightly regulated telomere maintenance (Hapangama et al., 2017). Disruption of telomere homeostasis not only aids oncogenesis but is also associated with reproductive ageing, infertility, and tissue dysfunction (Hapangama et al., 2017). Thus, telomeres constitute a biological crossover point between cancer development and reproductive health in gynaecological tissues (Fan et al., 2021). This review maintains a deliberate distinction between normal telomere physiology in reproductive contexts, which serves tissue homeostasis and fertility, and the pathological telomere dysfunction that drives tumor biology. Proposed links between reproductive aging and carcinogenesis are noted where supported by oncologic data but are otherwise flagged as speculative or context-dependent, to avoid conflating physiological telomere dynamics with carcinogenic mechanisms.

Growing evidence supports the clinical relevance of telomere length, telomerase activity, and alternative lengthening of telomeres as key clinical biomarkers for estimating cancer risk, prognosis, and response to therapy (Loukopoulou et al., 2024). However, the clinical relevance of these parameters remains variable and inconsistent across gynecological cancer subtypes, with most supporting studies being observational and lacking standardized methodology.

Intervention strategies targeting telomere dysfunction have expanded beyond conventional anticancer therapies to encompass plant-derived and other bioactive compounds, as well as lifestyle modifications aimed at reducing oxidative stress, inflammation, and cellular ageing. This narrative review aims to synthesise available data on the mechanisms underlying telomere-related genomic instability in gynaecological cancers.

Although this review covers the full range of gynecological malignancies, the authors note that evidence on telomeres is not uniform across cancer types. Hence, we synthesise available scientific evidence on telomere-driven dysfunctional changes in cervical, endometrial, ovarian, vulvar and vaginal cancers while presenting a conceptual framework for shared mechanisms, knowledge gaps, and future research directions. We also review telomere-associated biomarkers and therapeutic, plant, and lifestyle interventions targeting telomere biology for ovarian, endometrial, cervical, vulvar, and vaginal malignancies. By integrating global epidemiological burden with knowledge at the molecular and translational levels, this review presents telomere dynamics in the development of gynaecological cancers to advance diagnosis and inform treatment strategies.

## 2 Fundamentals of telomere biology in cancer

Telomeres are recurring DNA-protein complexes that protect the integrity of the linear chromosomes. They are tandem (TTAGGG) in repeats, which in humans are bound by the six-protein shelterin complex-TRF1, TRF2, TIN2, POT1, TPP1, and RAP1-organizing the telomeric DNA into a T-loop structure (de Lange, 2018). This architecture keeps the chromosome ends undetected as DNA double-strand breaks and inhibits the improper activation of DNA damage response (DDR) pathways, including ATM and ATR (Palm and de Lange, 2008; Sfeir and de Lange, 2012). In addition to end protection, telomeres regulate chromosome segregation and nuclear organization (Pfeiffer and Lingner, 2013). Importantly, in female reproductive tissues, these roles extend to the maintenance of ovarian reserve and endometrial function, both of which are critically dependent on chromosomal stability for gametogenesis, implantation, and sustaining pregnancy.

Telomeres also shorten with each cell division as a result of the end-replication problem and oxidative stress (Shay and Wright, 2019). Critically short telomeres lose shelterin-mediated capping function and produce a DNA damage response (DDR) signal that activates replicative senescence or apoptosis *via* p53 and in p16^INK4a^/Rb pathways (Victorelli and Passos, 2017). In premalignant lesions however, the cells can evade senescence checkpoints, leading to a telomere crisis of unchecked chromosomal instability and breakage -fusion -bridge cycles (Maciejowski and de Lange, 2017; Pampalona et al., 2010). Although crisis is usually fatal, rare clones stabilize telomere length through activation of telomere maintenance mechanisms (telomerase or ALT), thereby acquiring replicative immortality. Evidence on the role of telomere biology in the development of gynaecological cancer types is still fragmented and inconsistently translated into clinical settings (Loukopoulou et al., 2024; Hapangama et al., 2017). Their inclusion is therefore meant not to imply equal weight as evidence, but to outline a conceptual framework.

Telomerase reactivation is the most common pathway that is seen in 85%–90% of human cancers (Kim et al., 1994; Shay and Wright, 2019). Telomerase reactivation stabilises telomeres and enables continued cellular proliferation beyond normal replicative limits (Horn et al., 2013; Akincilar et al., 2016; Greider, 2016). Notably, telomerase also has extratelomeric activity, such as Wnt/β-catenin signaling regulation, mitochondrial metabolism, DNA repair, and transcriptional regulation of gene expression (Choi et al., 2008). Of particular relevance to gynaecological tissues, Mordechai et al. (2020) showed that non-canonical TERT controls the expression of steroidogenic genes and promotes production of steroid hormones in granulosa cells implicating non-canonical TERT activity in ovarian follicular function and female reproductive biology. These non-canonical functions further enlarge the oncogenic repertoire of TERT, and may be important in the initiation of tumor development independent of telomere elongation. Physiologically, however, transient telomerase activity is required in the female germline and the cycling endometrium, where it promotes follicular development, embryo implantation, and early placentation (Tanaka et al., 1998). Dysregulated telomerase, therefore, carries a dual consequence: it sustains tumor growth but, when deficient, it undermines fertility potential.

**FIGURE 1:**
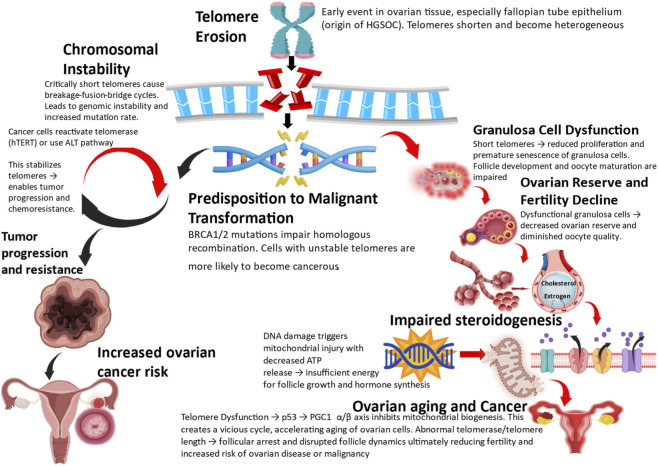
Telomere dysfunction in ovarian tissues. Telomere length is inherently heterogeneous, as each chromosome carries its own distinct telomere length. This figure depicts the stepwise consequences of telomere dysfunction in ovarian tissues that start with telomere erosion and chromosomal instability, triggering genomic vulnerability, especially in the context of the disruption of BRCA1/2 genes. From this shared early pathway, telomere dysfunction leads into two distinct and parallel ways: a cancer pathway, where the telomerase reactivation, or ALT, maintain the telomere stability to allow the progression and chemoresistance of the tumor, and an ovarian dysfunction pathway, where impaired granulosa cell senescence, impaired steroidogenesis, mitochondrial dysfunction, and energy depletion lead to accelerated ovarian aging and reproductive dysfunction. *Figure was created using canva, Biorender and MS Powerpoint tools.

A minority of cancers (about 10–15 percent) follow the alternative lengthening of telomeres (ALT) pathway, which is an independent telomerase-based process that is mediated by homology-directed repair of telomeric DNA. ALT is often linked with either ATRX or DAXX dysfunction and it is characterized by recombination-mediated telomere elongation and telomere length heterogeneity (Heaphy et al., 2011; Lovejoy et al., 2012). The activation of ALT is common in mesenchymal and neuroepithelial tumors and has also been observed in subsets of gynecological tumors (Bryan et al., 1995). Even though the mechanisms of action of ALT are less evidently related to reproduction, ALT activity was detected in the pre-implantation embryo (Varela et al., 2011). Moreover, it has been indicated that uncontrolled recombination of telomeric ends can impair meiotic fidelity in oocytes, thereby linking ALT-related instability to diminished reproductive competence (Tire et al., 2024) (Figure 1).

Telomere dysfunction is therefore a two-edged sword in oncogenesis. Telomere shortening mimics a tumor suppressive response that prevents unchecked proliferation, but critically short telomeres (end-to-end chromosome fusions, anaphase bridges, chromosome breaks) are the major causes of chromosomal instability that promotes malignant transformation when cells avoid senescence by reactivating telomerase or activating ALT (Baird, 2008; O’Sullivan and Karlseder, 2010; Maciejowski and de Lange, 2017). The same duality finds its reflection in female reproductive biology: on the one hand, the shortening of telomeres limits excessive cell proliferation, while, on the other hand, it also drives reproductive aging, infertility, and adverse pregnancy outcomes. Conversely, high telomerase activity increases cancer risk in reproductive tissues (Telfer et al., 2005). Maintaining balanced telomere dynamics is therefore essential for both oncologic protection and reproductive health. One of the main unresolved questions in telomere biology is whether telomere dysfunction is the determinant of gynecological carcinogenesis or a consequence of malignant transformation. Its effect is highly context- and stage-specific, as it represents a balance between tumor-suppressive and tumor-promoting forces.

Mechanistic clarity requires distinguishing telomere shortening as a driver of carcinogenesis from shortening as a consequence of it. In a normal cell, shortening of telomeres triggers entry into senescence to prevent cancer through the activation of p53/p16INK4a-Rb pathway (Wang et al., 2013). However, in premalignant cells with defective checkpoints, the endpoints of shortened telomeres can act as drivers of cancer by activating DNA damage response *via* ATM/ATR kinases, inducing breakage-fusion-bridge (BFB) cycles and generating complex chromosomal rearrangements that drive genomic instability and subsequent clonal evolution (Maciejowski and de Lange, 2017). Two mechanisms of escape from the crisis state of premalignant cells have been defined, which are different in gynaecological cancers. Telomerase reactivation to malignant immortality *via* TERT reactivation, especially through TERT promoter mutation, c-Myc amplification and/or HPV E6 inactivation of p53, predominantly occurs in cervical and endometrial cancers (Li and Tergaonkar, 2016). In contrast, ALT route to immortalisation is predominantly associated with clear cell and endometrioid ovarian cancers and is mediated by ATRX/DAXX loss (Li et al., 2019). Understanding this distinction is crucial for the meaningful interpretation of biomarkers and for selection of appropriate targeted therapies.

In addition to its role as a crucial determinant for telomere stability in cancer cells, the activity of telomerase in the stem cells of adult tissues has recently received attention. It has been established that telomerase is selectively expressed in stem cells of many adult tissue types, enabling them to maintain their telomeres and multiply for generations without losing telomere length (Cong et al., 2002). For gynaecological tissues, such as the endometrium and ovary, telomerase is required for the stem/progenitor cells that maintain tissue homeostasis and allow cyclical regeneration of the tissue (Valentijn et al., 2013). Interestingly, hTERT also has been reported to induce cancer stem cell (CSC) like properties in ovarian cancer cells (Valentijn et al., 2013). Stem cells have been characterized in endometrial tissues and show reduced marker expression and reduced telomerase activity in endometrial polyps compared to normal endometrium (Valentijn et al., 2013). Endometrial stem cell pathology thus suggests that an early role for dysregulated telomerase expression in stem cell populations could be involved in the carcinogenesis of gynaecological cancers (Hapangama et al., 2017). The inadequate focus of telomerase on the stem cell compartment provides an underappreciated view of how telomere biology can contribute to gynaecological carcinogenesis.

In addition to the instability of telomeres and their roles in carcinogenesis, the telomeric repeat-containing long non-coding RNA TERRA also has been studied in cancer biology. TERRA is a long non-coding RNA transcribed from sub-telomeric regions proximal to chromosome ends, and forms telomere heterochromatin and plays roles in maintaining telomere integrity and responding to DNA damage (Bettin et al., 2019). TERRA and its stability are altered during carcinogenesis, including in gynaecological cancers (Oh et al., 2017; Adishesh et al., 2020). TERRA species in cervical cancer cells exhibit variable abundance and stability among different cell lines but generally increased compared with HeLa cells (Oh et al., 2017). However, the abundance of chromosome-specific TERRA elements, especially those transcribed from 16p and 20q in endometrial cancer are decreased compared with healthy endometrium (Adishesh et al., 2020). TERRA in endometrial carcinogenesis is attenuated and inversely correlates with proliferation marker Ki67, indicating a possible tumour-suppressive or homeostatic role in endometrial cancer (Kitson et l., 2017). The study of TERRA in cancer is an emerging field and requires further investigation as potential biomarkers and therapeutic targets in gynaecological malignancies.

## 3 Telomere alterations in gynecological malignancies

### 3.1 Ovarian cancer

Telomere erosion is an early hallmark of ovarian carcinogenesis, particularly in the fallopian tube epithelium, the proposed origin of high-grade serous ovarian cancer (HGSOC) (Labidi-Galy et al., 2017). However, given the majority of evidence is from cross-sectional analysis of surrogate tissues, it is unclear whether telomere shortening is a factor in malignant transformation, or a consequence of early genomic instability. The shortening of telomeres and their heterogeneity to a critical level causes chromosomal instability *via* breakage-fusion-bridge cycles, which increase the rates of genomic changes and tumor formation (Kuhn et al., 2012; Choi et al., 2008). Homologous recombination in BRCA1/2 mutation carriers is defective, which increases genomic instability caused by telomere dysfunction and also predisposes cells to malignant transformation (Apostolou and Fostira, 2013).

To escape replicative senescence, ovarian cancers re-express telomerase (Uruski et al., 2021). Almost all developed HGSOC are characterized by high levels of hTERT expression and telomerase activity, which is facilitated by c-Myc and the epigenetic changes in the hTERT promoter (Akincilar et al., 2016). Telomerase activation is a common feature of most cancers, including cancers with defects in homologous recombination, such as those oncogenes with BRAC1/2 mutations. BRCA1/2 mutations disrupt telomere homeostasis and are involved in genomic instability, and although tumor cells with sustained proliferation capacity often reactivate telomerase to maintain telomere length, there is no definite evidence of the unique elevation of telomerase in BRCA-mutated tumor cells or evidence that telomerase acts directly to override HR defects and induce tumor aggressiveness (Uziel et al., 2016). High telomerase activity in HGSOC has been linked to poor prognosis and chemoresistance in multiple cohorts; however, the prognostic importance of telomerase activity is not homogeneous, and conflicting findings differ by disease stage, treatment exposure, and the analytical methods used to evaluate telomerase function (Pompili et al., 2017).

Though telomerase reactivation is predominant, some of the ovarian cancers use the alternative lengthening of telomeres (ALT) pathway. ALT is typified by recombination-mediated telomere maintenance, ultralong telomeres and ALT-related PML bodies, frequently linked with ATRX/DAXX changes (Heaphy et al., 2011). It has been characterised as ALT activity, especially in clear cell and endometrioid ovarian carcinomas. This heterogeneity in telomere maintenance across ovarian cancer subtypes means these tumors can exhibit different biological behaviors and treatment susceptibility. The histone H3.3 telomeric repeats is deposited by ATRX/DAXX chromatin remodelling complex and loss of ATRX/DAXX leads to disruption of heterochromatin at telomeres and induction of homology-directed repair-based telomere elongation (ALT) (Heaphy et al., 2011; Lovejoy et al., 2012). The status of ALT can be inferred by the presence of specific markers, although these are not exclusive to ALT. The C-circle assay detects extrachromosomal circular telomeric DNA specifically enriched in ALT cells. The presence of ALT-associated PML bodies (APBs) can be visualized by co-immunofluorescence of PML and telomere-specific probes (Chen et al., 2021). Cells that have entered ALT show heterogeneous telomere length. The role of ALT in gynaecological malignancies is under-explored, but emerging studies suggest that the majority of ALT-positive tumors are inherently resistant to telomerase inhibition. Interestingly, suppression of telomerase upregulates ALT, and thus it remains important to determine predictive markers of telomerase suppression-induced replication stress (Muoio and Fouquerel, 2025). ALT-positive tumors also have elevated replication stress at telomeres and are selectively vulnerable to ATR, CHK1 and FANCM inhibitors, which act through mechanisms of synthetic lethality (Carson and Flynn, 2023). The prevalence of ALT-positive tumours in various gynaecological cancer subtypes and the utility of C-circle or APB-based assays as prognostic/predictive tumour stratification tools in cancer need to be explored further.

Telomere dysfunction has profound effects on ovarian function through multiple mechanisms. Critically shortened telomeres and reduced telomerase activity in ovarian granulosa cells impair their proliferative capacity and induce premature cellular senescence (Péntek et al., 2023). Since granulosa cells support follicle development and oocyte maturation, their dysfunction results in a decreased ovarian reserve, diminished oocyte quality, and compromised fertility (Harada, 2024). Telomere shortening is strongly associated with premature ovarian failure (POF) and premature ovarian insufficiency (POI), in which telomerase activity is reduced, and follicle depletion occurs more rapidly (Butts et al., 2009; Xu et al., 2017).

Telomere dysfunction is also known to disrupt ovarian steroidogenesis (Mordechai et al., 2020; Kim, B. et al., 2021) (Figure 1). Granulosa cells undergo massive proliferation during follicular development to continue producing steroid hormones, primary estrogens and progesterone, necessary for reproductive function (Schütz and Batalha, 2024). Reduced telomerase activity disrupts granulosa cell proliferation and induces premature apoptosis, reducing expression of steroidogenic enzymes and estrogen synthesis (Harada, 2024; Péntek et al., 2023). On the other hand, it has been established that overexpression of the telomerase catalytic subunit (hTERT) enhances steroidogenic gene expression and increases estrogen levels in granulosa cells. Estrogens themselves induce the activation of telomerase, and this creates a positive feedback loop in processes that are known to promote follicle maturation and steroidogenesis (Mordechai et al., Telomere dysfunction has been associated with increased oxidative stress and DNA damage and may contribute to mitochondrial dysfunction in ovarian cells. Impaired mitochondrial biogenesis and reduced ATP production can, in turn, limit the energy required for follicular growth and hormone production (Sahin et al., 2011). The telomere-p53-PGC1α regulatory axis is of key importance, wherein telomere depletion causes p53 activation, which inhibits PGC1α/β and mitochondrial biogenesis, leading to a detrimental vicious cycle, where ovarian ageing is accelerated (Sahin et al., 2011).

In the context of reproductive ageing, as well as pathologies such as polycystic ovary syndrome (PCOS), disordered regulation and elongation of telomeres have been implicated in the abnormal follicle dynamics that may lead to failure of ovulation and increase the risk of malignancy. Taken together, telomere dysfunction impairs ovarian physiology by reducing cell replication, compromising mitochondrial function, impairing steroidogenesis, and affecting the ovarian microenvironment, impairing fertility and contributing to ovarian disease.

### 3.2 Endometrial cancer

Endometrial cancer (EC) is a heterogeneous group of cancers that can be divided by molecular classifications, such as The Cancer Genome Atlas (TCGA) system and ProMisE system, of which roughly a quarter to a third will belong to the hypermutated mismatch repair deficient (dMMR) and the high microsatellite instability (MSI-H) groups (Talhouk and McAlpine, 2016). The highest burden in this molecular category is frequently driven by hypermethylation of the MLH1 promoter. It is also associated with a high mutational burden, immune cell infiltration, and sensitivity to immune checkpoint inhibitors (Cancer Genome Atlas Research Network et al., 2013).

In addition to their canonical roles in correcting base-mismatches and insertion-deletion loops during microsatellite instability, MMR proteins play significant roles in telomere-end protection. In preclinical models, MSH2, MSH6 and PMS2 proteins repress aberrant telomeric repeat recombination, and hence, telomere instability is prevented (Beishline and Azizkhan-Clifford, 2015). Telomeres in dMMR have enhanced sister-chromatid exchange, recombination and structural susceptibility to chromosomal rearrangements, which may hasten chromosomal instability and malignant progression (Alnafakh et al., 2019). While few functional studies specifically focus on endometrial carcinoma, dMMR likely fosters an environment of telomeric dysfunction in MSI-H EC (Alnafakh et al., 2019). At present, this link remains largely inferential, underscoring the need for mechanistic studies that directly interrogate telomere dynamics in molecularly stratified endometrial tumors. This telomere instability is not only crucial for carcinogenesis but also has consequences for endometrial regenerative capacity, since recurrent cycles of proliferation and differentiation depend on intact telomere maintenance.

Interestingly, the most common telomere maintenance process in EC is telomerase activation through transcriptional upregulation of the catalytic component hTERT, but the mechanisms driving this upregulation are strikingly subtype-specific. TERT promoter hotspot mutations (C228T and C250T) are detected in only approximately 5%–10% of endometrial cancers overall, concentrated in the copy-number-high (CNH), TP53-mutant subtype and in POLE-ultramutated tumours where the hypermutator phenotype increases the probability of acquiring promoter mutations (Praiss et al., 2023). By contrast, TERT promoter mutations are essentially absent in dMMR/MSI-H endometrial tumours, in which telomerase is reactivated through epigenetic mechanisms—principally *via* transcriptional de-repression of hTERT—rather than through genetic alteration of the TERT promoter (Alnafakh et al., 2019). Across gynaecological cancer subtypes, TERT promoter mutation frequencies vary considerably: mutations are rare in cervical cancer; they occur at low frequency in ovarian carcinomas (approximately 4%–6%), enriched in clear cell and mucinous histotypes; and are uncommon in vulvar and vaginal cancers (Praiss et al., 2023). These frequencies are substantially lower than in glioblastoma (∼80%) or melanoma (∼70%), cited only as external context; the clinically relevant variation is subtype-specific within gynaecological cancers. TERT-promoter-mutant tumours sustain constitutively elevated telomerase and may show differential sensitivity to telomerase inhibitors compared with epigenetically activated counterparts, while MSI-H tumours require epigenetic detection strategies. These distinctions underscore the need to integrate TERT mutation status with TCGA molecular subtype classification when designing telomerase-directed trials in EC. TERT promoter mutations are relatively rare compared to many other malignancies. In endometrial cancer, they occur in a small minority of cases (∼2–5%) and are enriched in copy-number-high (TP53-abnormal) tumors. In ovarian carcinoma, they are generally uncommon and variably reported, with possible enrichment in clear cell subtypes. In cervical cancer, TERT promoter mutations are infrequent, as telomerase is instead predominantly reactivated through HPV E6–mediated transcriptional upregulation (Annunziata et al., 2018; Praiss et al., 2023). This subtype-specific profile of TERT mutation *versus* non-mutational telomerase activation has direct translational implications in that TERT-promoter-mutant tumors may sustain telomerase at very high levels and may respond differently to telomerase inhibitors than epigenetically activated counterparts. Integrating TERT mutation status with TCGA molecular classifications, including the POLE-ultramutated, MSI-H, copy-number-low, and copy-number-high endometrial subtypes, as well as ovarian histotypes (HGSOC, clear cell, endometrioid), would sharpen both biomarker selection and therapeutic targeting. The immunogenic profile of dMMR/MSI-H tumors has been used therapeutically through PD-1 inhibitors, including dostarlimab and pembrolizumab, that have now been approved in the advanced or recurrent EC setting. However, telomere-based therapies are still largely experimental here (Zhou et al., 2025). Given that normal endometrial physiology requires dynamic regulation of telomerase across the menstrual cycle, dysregulation in MSI-H EC may interfere with hormone-driven tissue renewal and receptivity, potentially explaining altered implantation environments observed in premalignant or hyperplastic endometrium.

The human endometrium is a unique physiological model of steroid-regulated telomerase activity changing throughout the menstrual cycle. The proliferative phase, which is the estrogen-dominated phase, has been shown to increase telomerase activity, which favours cellular growth and telomere elongation; the secretory phase, progesterone lowers telomerase activity, limiting replication and leading to differentiation (Kim et al., 1994; Kyo et al., 1999). At the molecular level, estrogen increases hTERT expression by activating ERα (estrogen receptor alpha)-mediated transcription and downstream MAPK/ERK signalling cascades (Misiti et al., 2000). The mechanism increases replicative potential and telomere stabilisation, which, in the malignant environment, can strengthen tumor development. Progesterone, in contrast, inhibits telomerase by suppressing transcription of hTERT, a phenomenon observed in primary endometrial epithelial cells and EC-derived cell lines (Kim et al., 1994; Kyo et al., 1999). These mutually opposing regulatory events are clinically pertinent: progestin therapy, commonly used in fertility-sparing treatment of early-stage or low-grade EC, exploits the progesterone-mediated suppressive effect on telomerase activity and inhibits malignant proliferation (Emons et al., 2000).

**FIGURE 2:**
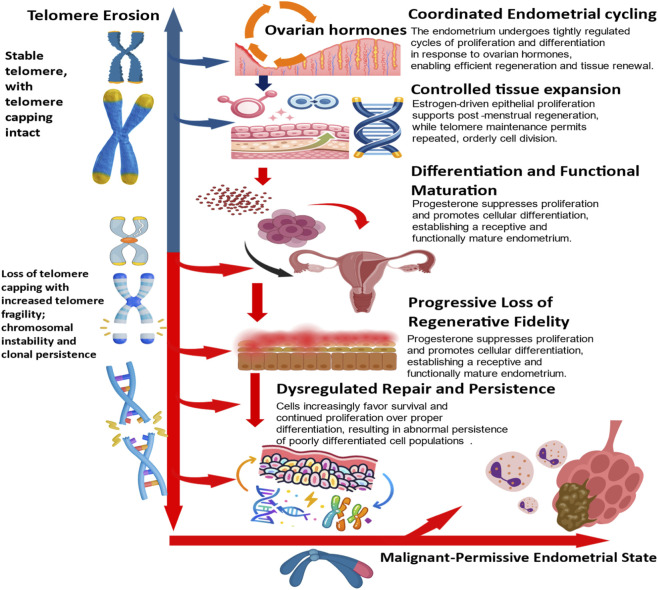
Telomere dysfunction in endometrial cancer. The figure illustrates a continuum in which progressive deterioration of telomere integrity is directly related to deterioration of endometrial function. In a normal tissue-renewing state, with intact, well-protected telomeres, the endometrium can cycle through repeated rounds of proliferation and differentiation in an organized, hormone-controlled way to renew tissues in the function of the cycle. As telomere capping is gradually lost, telomeres become fragile and exhibit structural heterogeneity, leading to replicative stress and low-grade defects in tissue regeneration. Over time, these changes disrupt the balance between proliferation and differentiation, and the endometrium undergoes inefficient repair, leading to increasing structural disorganization. At more severe stages of telomere dysfunction, chromosomal instability and clonal persistence (the sustained propagation of telomere-dysfunctional cells that have escaped senescence checkpoints) predominate, leading to prolonged proliferation, loss of differentiation, and a tissue environment permissive for malignant transformation. *Figure was created using canva, Biorender and MS Powerpoint tools.

Several mechanisms are involved in understanding the effects of telomere dysfunction during carcinogenesis and endometrial physiological processes. One mechanism is telomerase activity, which is regulated by hormones. Estrogen activates hTERT through ERa/MAPK/ERK signaling, which stimulates cellular proliferation and telomere elongation, while progesterone inhibits hTERT, thereby inhibiting telomerase activity and promoting differentiation in preparation for implantation (Kim et al., 1994; Misiti et al., 2000). Disruption of this balance skews the cycle between regeneration and receptivity, with implications for both fertility and tumorigenesis. Another mechanism is dMMR/MSI-H-driven telomere instability, in which MMR protein loss promotes telomeric recombination and fragility, leading to failure of chromosomal stability and reduced endometrial regenerative capacity (Beishline and Azizkhan-Clifford, 2015) (Figure 2. In addition, oncogenic pathway crosstalk contributes to telomerase dysregulation. PTEN loss potentiates PI3K/AKT activity on hTERT and reduces progesterone inhibition and Wnt/beta-catenin dysfunction, further enhancing hTERT transcription (Zhou et al., 2025; Lu and Broaddus, 2020). These changes affect the cyclical restructuring of the endometrium and, hence, receptivity and fertility outcomes. A further mechanism is that MSI-H tumors rely on epigenetic and transcriptional upregulation of hTERT rather than on promoter mutations that may antagonize the telomerase regulation in the endometrial monthly cycle, which is necessary for a functional endometrium. Clinically, aberrant telomerase activity has been linked not only to malignant progression but also to infertility (Hapangama et al., 2017).

A further level of complexity is imposed by cross-talk between hormone signaling and oncogenic pathways. Dysregulation of PI3K/AKT signaling, as with PTEN loss (a hallmark in EC), may sensitize hTERT expression and telomerase activation and antagonise progesterone-mediated suppression (Lu and Broaddus, 2020). Similarly, dysregulation of the Wnt/beta-catenin pathway has been shown to promote hTERT transcription; thus, this pathway links molecular defects commonly found in EC with abnormal telomerase regulation (Zhou et al., 2025). These changes not only promote carcinogenesis but also potentially impair endometrial receptivity and fertility success by disrupting the balance in cyclic endometrial remodeling, driven by altered proliferation and differentiation.

Telomere biology offers opportunities for biomarkers and targeted therapy in EC. Shortened telomere length and increased hTERT expression are correlated with higher tumor grade and a poor prognosis (Bujnak et al., 2025). Significantly, in the non-malignant setting, changes in telomerase activity are also linked to certain disorders of endometrial function, such as infertility, recurrent implantation failure, and endometriosis, suggesting overlap in telomere dysregulation between malignant and non-malignant lesions. Further enforcing the molecular complexity of EC, telomeric repeat-containing RNA (TERRA) has been found to be strongly decreased in endometrial carcinoma in comparison to healthy endometrium, with chromosome-specific TERRA species to be negatively correlated with a proliferative marker, Ki67, suggesting a potential tumor-suppressive function for TERRA in the endometrium (Adishesh et al., 2020). This finding highlights the significance of taking TERRA as a relevant dimension of telomere biology in endometrial cancer in addition to telomere length and telomerase activity. Telomerase inhibition remains experimental but promising therapeutically. First-in-class inhibitors, such as imetelstat, and nucleoside analogues, such as 6-thio-2′-deoxyguanosine (6-thio-dG), which induce telomere dysfunction, are under investigation in solid tumors and may apply to telomerase-active EC (Shay and Wright, 2019). Given telomerase’s role in normal endometrial renewal and receptivity, therapeutic strategies must carefully balance anti-tumor efficacy with preservation of residual endometrial function, especially in premenopausal women seeking fertility preservation [hTERT-based vaccines INVAC-1 and UV1 are discussed in the therapeutics section].

### 3.3 Cervical cancer

Telomere dysfunction in the pathogenesis of cervical cancer is well documented in the scientific literature, and evidence indicates roles in HPV-induced hTERT activation, shortening of telomeres in early lesions, gradual telomere shortening and stabilization, and clonal heterogeneity. The discussion section will elaborate on this mechanism, as it is not a prevailing model but rather a well-defined reference point that places any telomere-related process in other gynecological malignancies in context.

The causative agents of more than 90 percent of cervical cancer cases in the world, high-risk human papillomavirus (HPV) types, especially HPV16 and HPV18, make cervical cancer a paradigmatic model of virus-mediated oncogenesis in gynecologic cancer (de Martel et al., 2017). The HPV oncoproteins E6 and E7 are the main agents in activating telomerase, which is among HPV’s important oncogenic activities.

Transcription of hTERT is directly triggered by HPV E6 in the presence of c-Myc cellular transcription factor and other cofacters at the TERT promoter which results in persistent telomerase expression (McMurray and McCance, 2003; Kim et al., 2019). E6 is also involved in the epigenetic remodelling of the hTERT promoter, induced by histone modifications and DNA methylation, which aid in stabilizing transcriptionally upregulated hTERT (Kim et al., 2019). E7 goes hand in hand with this process by suppressing the retinoblastoma protein (pRb) pathway and disrupting cell cycle regulators, thereby providing a permissive environment for continuous telomerase activation and cell immortalization (Gabet et al., 2008). A combination of these viral oncoproteins transforms temporarily infected cervical epithelial cells into cells with an indefinite replicative potential - one of the hallmarks of malignant transformation. Clinically, hTERT expression has been used as a replacement to signify excessive HPV oncogenic activity, and elevated hTERT expression in persistent HPV infection, cervical intraepithelial neoplasia (CIN), invasive carcinoma, and lesions with a poor prognosis (Wang et al., 2024).

The development of cervical carcinogenesis is progressive and starts with low-grade CIN through high-grade CIN to invasive carcinoma. Telomere shortening can be observed in cervical epithelial cells at early stages, occurring in part because of replicative stress, chronic inflammation, and the expression of viral oncogenes (Stöppler et al., 1997). This is one of the reasons for chromosomal instability, including end-to-end chromosomal fusions, aneuploidy, and, consequently, an increased risk of oncogenic transformation. The regenerative balance of the cervical epithelium is compromised by dysfunctional telomere length, thereby interfering with basal and parabasal layer turnover, barrier strength, tissue repair, and local immunosurveillance. The alterations provide a microenvironment that favors the clonal growth of malignant cells.

The lesions progress to activate hTERT, stabilizing telomeres, counteracting the initial telomere crisis, and preventing cells from evading senescence or apoptosis (Klingelhutz et al., 1996; Meena et al., 2015). This telomere stabilization, favoring the production of driver mutations on oncogenes and tumor suppressors, triggers a gradual transition of CIN to invasive carcinoma. Telomere shortening, followed by telomerase reactivation and re-stabilization, is typical of cervical tumorigenesis. Notably, invasive cervical carcinomas exhibit heterogeneity in telomere length, with telomere shortening in one area followed by stabilization and/or elongation of adjacent telomeres, a manifestation of clonal selection during tumor evolution (Meena et al., 2015).

**FIGURE 3:**
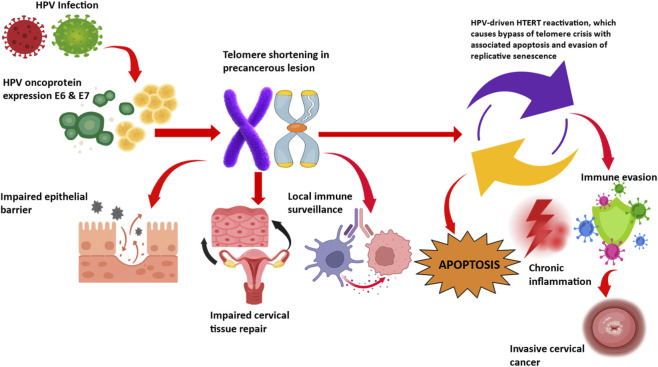
Mechanistic illustration of telomere-driven cervical carcinogenesis. Cellular stressors such as viral oncogene activity accelerate telomere attrition in cervical epithelial cells. The sustained shortening of telomeres compromises chromosomal end protection, leading to genomic instability, chromosome fusions, and disrupted cell-cycle control. This also disrupts normal tissue homeostasis, impairing epithelial turnover, barrier integrity, and immune surveillance, thus creating a permissive microenvironment for clonal expansion as telomere dysfunction worsens. Subsequently, the emerging neoplastic cells reactivate telomerase, thereby stabilizing critically short telomeres and enabling continued proliferation, bypassing telomere crisis and apoptosis despite accumulated genomic damage. This cycle of telomere shortening-reactivation causes clonal heterogeneity and tumor evolution, while telomere-associated senescence and inflammatory signaling further contribute to immune evasion, disease progression, and, eventually, malignant transformation. *Figure was created using canva, Biorender and MS Powerpoint tools.

Besides the role of dysregulated telomere biology in transforming a progenitor cell into a replicatively immortal cell, inflammatory signaling similar to the senescence-associated secretory phenotype (SASP) can still occur through persistent telomeric DNA damage signaling, regardless of full replicative senescence, and can contribute to immune evasion and tumor development. Furthermore, the central role of hTERT reactivation in malignant transformation highlights its potential as a therapeutic target in cervical cancer, providing a mechanistic basis for telomerase-targeted treatments (Figure 3).

### 3.4 Vulvar and vaginal cancers

Evidence of an association between telomere dysfunction and vulvar/vaginal carcinogenesis is scanty; therefore, this section presents available observational findings as well as biologically plausible mechanisms, instead of definitive causal models.

In vulvar squamous cell carcinoma (VSCC), telomeres are often shortened to a critical level in precancerous lesions (vulvar intraepithelial neoplasia, VIN) and invasive tumors (Hoang et al., 2016). Shortened telomeres lead to chromosomal instability, which, in turn, causes abnormal cell division and loss of normal tissue architecture (Wellenhofer and Brustmann, 2012). Functionally, this is reflected in disturbed epithelial barrier function, epithelial ulceration, and tissue growth disorders, leading to impairments in the protective and sensory functions of the vulva. Tumor cells evade senescence by activating the telomerase enzyme (hTERT), which enables them to continue dividing despite DNA damage, further deteriorating tissue organization and normal vulvar function (Wellenhofer and Brustmann, 2012; Salama et al., 2022).

Vaginal intraepithelial neoplasia (VaIN) and vaginal invasive tumors are also associated with telomere shortening (Pańczyszyn et al., 2018). Dysfunctional telomeres trigger DNA damage responses and genomic instability, which disrupts normal vaginal epithelial renewal (Boniewska-Bernacka et al., 2024). As a result, tissue integrity is lost, leading to lesions, abnormal bleeding, and dysfunction of the vaginal mucosa. Overexpression of telomerase in these cells enables them to survive and grow uncontrollably, promoting tumor growth and potentially disrupting the sexual, protective, and reproductive functions of the vagina (van der Avoort et al., 2006).

**FIGURE 4:**
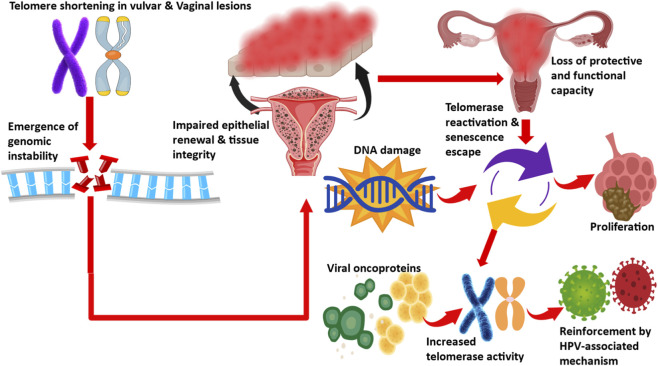
Telomere dysfunction and progressive vulvovaginal epithelial disruption. The mechanistic evidence supporting telomere dysfunction as a driver of vulvar and vaginal carcinogenesis is substantially less developed than for cervical or ovarian cancers, and readers should interpret the following discussion accordingly. Direct telomere-specific studies in these malignancies are sparse; much of the biological framework presented here is extrapolated from cervical cancer models or inferred from shared risk factors such as HPV infection and chronic inflammation. These sections are therefore best understood as hypothesis-generating, identifying plausible mechanisms that warrant dedicated empirical investigation rather than established biology. With that caveat, Telomere shortening is observed in vulvar and vaginal precancerous lesions and is maintained in invasive tumors, indicating progressive telomere loss in the development of the disease. Critically shortened telomeres induce chromosomal instability and abnormal cellular division, which disrupts epithelial architecture and compromises tissue renewal capacity. As instability continues, tissue integrity and barrier functions are reduced, leading to ulceration, abnormal growth, and loss of protective, sensory and secretory functions. To overcome senescence, tumor cells reinstate telomerase (hTERT) activity, allowing them to continue dividing despite ongoing DNA damage. However, viral oncoproteins further increase telomerase activity in HPV-associated lesions, reinforcing cellular immortalization, architectural disorganization, and progression of disease. *Figure was created using Canva, Biorender and MS Powerpoint tools.

Telomere dysfunction leads to a series of genomic instabilities, including end-to-end chromosome fusions and the accumulation of DNA damage (Cleal and Baird, 2020). In HPV-associated cancers, viral proteins, such as E6, further promote telomerase activity, increasing the rate of epithelial cell immortalization (Figure 4). The resulting uncontrolled cell proliferation and architectural disruption compromise the regular protective, sensory, and secretory roles of the vulvar and vaginal tissues, contributing directly to disease symptoms and progression (Yeo-Teh et al., 2018).

## 4 Clinical applications of telomere biology

### 4.1 Diagnostic application

From the diagnostic standpoint, there are several possibilities for making early detection and risk stratification of gynecological cancers based on telomere biology. Detection of the presence of telomerase activity and telomere length abnormalities in exfoliated cervical cells has been suggested as a complementary biomarker for the detection of high-grade cervical intraepithelial neoplasia and early detection of cancer (Meena et al., 2015). The progressive increase in hTERT expression along the CIN/cancer continuum further supports the increased relevance of this factor as a biomarker and a potential therapeutic target. Clinically, this is important not only from the oncologic standpoint, but also because overactivity of telomerase could cause perturbation of epithelial differentiation, which in turn could impair the barrier as well as secretory functions of the cervix, which also have critical importance for reproductive health. In addition, the telomeric non-coding RNA TERRA has been shown to undergo significant alterations in cervical cancer cells; its abundance and stability vary substantially across cervical cancer cell lines and are not simply linked to telomere length, suggesting that TERRA dysregulation constitutes an independent dimension of telomere biology in cervical carcinogenesis (Oh et al., 2017).

Assessment of telomere length in tumor tissues compared to adjacent non-neoplastic counterparts can reflect the degree of telomere attrition during carcinogenesis; however, average telomere length alone does not directly indicate genomic instability, since senescent cells with short telomeres halt proliferation as a protective mechanism. Clinically meaningful genomic instability arises when cells bypass senescence, not merely from telomere shortening *per se* (Mehrez et al., 2019). Pronounced telomere shortening in malignant cells has been described as a molecular hallmark of tumor initiation and progression, which contributes to chromosomal instability and altered cellular fate (Lin et al., 2013). However, although interest in telomere length as a diagnostic or prognostic biomarker has increased, the results of gynecological cancer studies are inconsistent. Reported associations are subject to a variety of factors, such as cancer type, tissue source, stage of disease, and method of analysis, and most studies are observational in nature, which limits causal inference and ability to translate findings to the clinic.

Although indirect, peripheral blood leukocyte telomere length represents a minimally invasive approach to assessing systemic telomere attrition and biological ageing, with shorter telomeres reported to associate with higher susceptibility to gynecological malignancies in some cohorts (Rode et al., 2015). Liquid biopsy platforms also pave the way for liquid biopsy analysis of circulating cell-free telomeric DNA, which holds possibilities to monitor the tumor burden in real time, telomere status and response to treatment (Tadimety et al., 2018; Sorbini et al., 2024). Nevertheless, interpretation of telomere length parameter information is complicated by large heterogeneity in methodology, e.g., differences between assays using qPCR-, TRF- or q-FISH-based techniques, and variation in whether telomere length is measured in tumor tissue, adjacent normal tissue, or peripheral blood leukocytes. As such, telomere length alone has not yet proven to be a definitive diagnostic marker.

## 5 Prognostic utility

However, telomere-related biomarkers can also have a possible prognostic relevance, although their added clinical value is context-specific. Tumors with significant telomere dysfunction, telomerase reactivation, or activation of alternative telomere maintenance mechanisms were found to be associated with malignant phenotypes, including higher recurrence rates, therapeutic resistance, metastatic potential, and poorer overall survival (Shay, 2014). Longitudinal evaluation of telomere dynamics over treatment years may offer additional clues and help further understand prognosis, since case status, treated and/or critically short telomere stabilization during treatment, could indicate treatment resistance and increased risk of relapse (Matsuda et al., 2023).

Importantly, there is increasing evidence that, for several reasons, telomere dysfunction, telomere maintenance mechanisms, and dynamic changes through time may be more biologically and clinically informative than absolute measures of telomere length alone. Accordingly, combining parameters related to telomere composition with currently known clinicopathological and molecular metrics could improve risk stratification and enable more personalized treatment in gynecological oncology, without overreliance on a single static biomarker (Chen et al., 2024).

## 6 Methodological limitations

Several methodological flaws limit interpretation of telomere results in gynaecological cancers. Telomere length measurements are highly assay-dependent, ranging from qPCR for relative measurements, terminal restriction fragment (TRF) analysis for absolute length distributions, and quantitative fluorescence *in situ* hybridisation (Q-FISH) for cell-specific resolution, with different strengths and inherent biases. Telomere length is also under strong influence of the age, systemic inflammation level, metabolic condition and past exposures to genotoxic therapies or telomerase inhibitors, and may thus confound associations attributed just to tumor biology. In addition to this, telomere dynamics vary greatly between different types of cells within the tumor microenvironment, and bulk tissue profiling might disguise clinically significant heterogeneity of populations of malignant, stromal and immune cells. It should also be noted that telomere length is inherently heterogeneous even within a single cell, as each chromosome carries its own distinct telomere length; thus bulk measurements represent an average that masks this fundamental biological variation. Epidemiologic studies using peripheral blood leukocyte telomere length as a surrogate for tumor telomere biology may therefore be misleading because of an inconsistent relationship with intratumoral telomere biology. This limitation is considered especially important since telomere dysfunction is not a simple function of telomere length, but is instead dependent on telomere capping, integrity of the shelterin complex, and DNA damage response signalling, such that maintaining the same telomere lengths in two tumors may lead to dissimilar biological behaviour by the two tumors. Moreover, most available studies are cross-sectional and do not allow inference of temporal changes in telomere length during disease progression or treatment. Although therapeutic approaches that target telomerase have been promising, the available evidence is preclinical, and their safety, efficacy and fertility implications in gynaecological cancers need to be validated in clinical trials designed accordingly. These factors contribute to substantial inter-study variability and currently limit the direct clinical translation of telomere-based biomarkers. Critical appraisal of these methodologies is essential for clinicians seeking to translate telomere-based findings. Among current approaches, qPCR is the most scalable but measures only relative telomere length and is highly sensitive to technical variation across laboratories. TRF analysis provides absolute telomere length distributions and captures heterogeneity but is labour-intensive and requires substantial tissue input. Q-FISH offers single-cell resolution and can detect telomere-free chromosome ends, making it the most biologically informative method for capturing true telomere dysfunction rather than simple length reduction; however, it is not yet feasible in routine clinical pathology workflows. Crucially, the distinction between telomere length and telomere dysfunction must be front and centre in interpreting results: two tumours with identical average telomere lengths may differ markedly in shelterin integrity, DDR activation, and malignant behaviour. Blood-based leukocyte telomere length has poor concordance with intratumoral telomere biology and cannot substitute for tissue-based approaches.

In terms of translational readiness, a tiered framework must specify clinical context—diagnostic, prognostic, or predictive—since the evidence base differs across applications. In the diagnostic context, tissue-based hTERT expression is most clinically advanced in HPV-driven cervical cancer, where prospective data report sensitivity of 70%–85% for high-grade CIN in tissue biopsy specimens (Wang et al., 2024; Meena et al., 2015). This utility does not extend to non-HPV-driven gynaecological cancers: physiological hTERT expression in the cycling endometrium and ovarian granulosa cells substantially reduces specificity in endometrial and ovarian contexts, and this limitation must be explicitly acknowledged when extending cervical frameworks to other subtypes (Kim et al., 1994; Hapangama et al., 2017). In the prognostic context, tumour tissue telomere length by Q-FISH—providing single-cell resolution and detecting telomere-free chromosome ends—has been associated with higher tumour grade and poorer outcomes (Meena et al., 2015; Lin et al., 2013); Q-FISH remains infeasible in routine pathology and sits at the discovery-to-validation transition. In the predictive context, no validated biomarker currently exists for telomerase-directed therapies in gynaecological cancers, though ALT status by C-circle assay or APB detection is an emerging candidate predictor of resistance. Blood-based leukocyte telomere length has poor concordance with intratumoral biology and cannot substitute for tissue-based approaches. A proposed tiered framework assigns: tissue-based hTERT in HPV-driven cervical cancer to the highest tier (approaching clinical validation trials); Q-FISH telomere length and TERT promoter mutation profiling to an intermediate tier (requiring prospective validation in molecularly stratified cohorts); and composite telomere dysfunction scores to an exploratory tier. Applying this subtype-specific framework is essential for meaningful translational progress.

Future studies using standardised, cell-resolved and longitudinal methods of telomere evaluation will be necessary to enhance reproducibility and elucidate the clinical significance of telomere-mediated dysfunction in gynecological cancers.

## 7 Approaches to therapeutic intervention

### 7.1 Telomerase inhibitors

Direct telomerase inhibition is a rational therapeutic approach in gynaecological malignancies, where the molecular hallmark of these tumors is telomerase reactivation. Oligonucleotide- and small-molecule-based inhibitors represent the main pharmacologic approaches currently being explored (Alnafakh et al., 2019). Among those, imetelstat (GRN163L), a 13-mer thiophosphoramidate oligonucleotide derivative attached to a lipid moiety, binds directly to the template region of the telomerase RNA component (hTR/TERC) and competes with telomere elongation (Alnafakh et al., 2019). Repetitive and ongoing exposure over several cell cycles leads to progressive telomere shortening and, eventually, to proliferative arrest, apoptosis, or mitotic catastrophe in telomerase-dependent cancer cells (Alnafakh et al., 2019).

Importantly, prior to initiating telomerase inhibitor therapy, tumour telomere maintenance status should be assessed, as ALT-positive tumours will be intrinsically resistant to telomerase inhibition and ALT activity may be further upregulated upon telomerase suppression. Preclinical models of cervical and ovarian cancer have shown that imetelstat inhibits clonogenic survival, increases chemosensitivity and inhibits xenograft tumor growth, and thus represents a potential tool in gynecologic oncology (Alnafakh et al., 2019). The therapeutic potential of telomerase inhibition is supported by its prevalence across gynaecological cancers. Given that over 85% of cervical, ovarian, and endometrial cancers exhibit telomerase reactivation, with upregulation of telomerase reverse transcriptase (TERT), targeting telomerase could provide a selective approach, noting the important caveat of potential toxicity to telomerase-positive stem cells (see also Major Comment; telomerase activity in stem cells is discussed in the Fundamentals section) and activated lymphocytes (Schrank et al., 2018). In addition to telomere shortening, telomerase inhibition results in telomere dysfunction, corresponding DNA damage responses, with the appearance of critically short telomeres being identified as double-strand breaks, which trigger ATM/ATR-p53 signaling pathways, leading to senescence or apoptosis.(16) In addition to this, inhibition of non-canonical telomerase functions, including, for example, involvement in Wnt/beta-catenin signaling pathways and epithelial-mesenchymal transition, further limits invasion and metastatic potential (Gu et al., 2024).

### 7.2 Clinical challenges: Toxicity and hematopoietic effects

Despite their mechanistic appeal, telomerase inhibitors face significant translational barriers, most notably dose-limiting hematopoietic toxicity (Lupatov and Yarygin, 2022). Telomerase activity is physiologically maintained in hematopoietic stem and progenitor cells (HSPCs) to preserve telomere integrity and sustain lifelong self-renewal (Lupatov and Yarygin, 2022). Pharmacologic inhibition disrupts this balance, inducing telomere attrition, DNA damage signaling, chromosomal instability, and eventual apoptosis or senescence of progenitor populations (Vasko et al., 2017). Clinically, this manifests as cytopenias, of which neutropenia, thrombocytopenia, and anaemia have been consistently reported in early-phase trials with imetelstat (Vasko et al., 2017).

One of the main limitations of therapeutic use stems from the time-dependent nature of these effects: Antitumor efficacy correlates with cumulative telomere shortening over weeks, while the marrow-suppressive effects emerge earlier due to rapid cycling of HSPCs (van den Boogaard et al., 2022). This problem is further aggravated by the use of telomerase inhibitors in combination with platinum compounds or taxanes, which are widely used in gynecologic oncology, thereby increasing bone marrow toxicity (van den Boogaard et al., 2022). Other off-target effects might include gastrointestinal mucositis, increased hepatic enzymes, and the inability to expand lymphocyte clones, which would prevent use in combination with immunotherapeutic methods (Huang et al., 2024).

Another problematic area of clinical translation is patient heterogeneity. Those with low baseline marrow reserve or short leukocyte telomeres may be more prone to toxicity, whereas tumors with alternative lengthening of telomeres (ALT) may be intrinsically resistant (Yang et al., 2017). Some of the mitigation approaches under development include intermittent dosing schedules, intensive hematologic monitoring, growth factor use, patient selection based on biomarkers, and tumor-targeted oligonucleotide delivery systems aimed at reducing systemic exposure (Jain, 2017). The most common obstacle to using telomerase inhibitors to treat gynecologic cancer is balancing between the long-term efficacy and the tolerability of the treatment. The temporal dimension of telomere dysfunction is an underappreciated yet clinically significant aspect of gynaecological carcinogenesis. In cervical cancer, cells first enter crisis due to telomere shortening, then upregulate HPV E6 to induce TERT transcription, rescuing them from crisis; the same sequential logic applies in endometrial and ovarian cancers, where premalignant cells undergo iterative cycles of crisis, escape, and clonal selection. Understanding how treatment modalities interact with telomere biology is increasingly critical to predicting and overcoming resistance.

### 7.3 Platinum-based chemotherapy

Platinum-based chemotherapy, the backbone of ovarian cancer treatment, generates interstrand crosslinks (ICLs) that are particularly damaging at G-rich telomeric repeat sequences, susceptible to ICL formation and oxidative modification (O'Sullivan and Karlseder, 2010). Platinum-sensitive clones with short telomeres undergo accelerated telomere dysfunction and apoptosis, while telomerase-high or ALT-active clones are selectively expanded; preclinical ovarian cancer data indicate that telomerase-high clones exhibit attenuated platinum sensitivity and that pharmacological telomere destabilisation can restore responsiveness, providing mechanistic support for telomere-mediated platinum resistance (Pompili et al., 2017; Uruski et al., 2021).

### 7.4 PARP inhibitors

PARP inhibitors (PARPi) interact with telomere biology through a distinct mechanism: PARP1 is recruited to deprotected single-stranded telomeric overhangs and promotes alternative end-joining (alt-EJ), driving end-to-end chromosome fusions and breakage-fusion-bridge cycles (Gu et al., 2024). In BRCA1/2-deficient tumours, PARP1-mediated alt-EJ becomes the predominant route by which telomere dysfunction propagates genomic instability; PARPi amplify this replication stress additively to their effects at non-telomeric DSBs (Gu et al., 2024; Fernandez et al., 2025), providing rationale for PARPi–telomerase inhibitor combinations in BRCA-mutant disease.

#### 7.5 Immune checkpoint inhibitors

Immune checkpoint inhibitors (ICIs), now standard in dMMR/MSI-H endometrial cancer, connect to telomere biology through MMR protein loss: dMMR promotes telomere instability and generation of neo-antigenic peptides from telomere-associated sequences and BFB-derived rearrangements (Alnafakh et al., 2019; Beishline and Azizkhan-Clifford, 2015). Whether telomere-driven neoantigen burden specifically predicts ICI response in MSI-H endometrial cancer remains undemonstrated; the co-occurrence of dMMR, telomere instability, high tumour mutational burden, and ICI sensitivity suggests telomere dysfunction may contribute to the immunogenic phenotype. Prospective telomere profiling in ICI trials would be a productive research direction. Assessment of telomere maintenance status at diagnosis, during therapy, and at relapse would provide real-time insights into treatment resistance.

### 7.6 Telomerase-based immunotherapy

Telomerase-based immunotherapy exploits the near-universal expression of hTERT in gynecological cancers to generate tumor-selective immune responses (Ellingsen et al., 2021). Peptide-, DNA-, RNA-, and dendritic cell–based vaccines encoding hTERT epitopes stimulate cytotoxic CD8^+^ and helper CD4^+^ T-cell responses through antigen presentation on MHC class I and II molecules (Thalmensi et al., 2016). Activated T cells mediate tumor cell killing *via* perforin–granzyme release, Fas/FasL signaling, and cytokine-driven cytotoxicity, while CD4^+^ T-cell activation supports dendritic cell priming and immunologic memory (Thalmensi et al., 2016).

Vaccines such as GV1001 and GX301 have demonstrated immunogenicity and antitumor activity in preclinical ovarian and cervical cancer models, offering the advantage of more rapid immune-mediated tumor control compared with the delayed effects of telomerase inhibition alone (Kailashiya et al., 2017). However, clinical efficacy is limited by tumor immune evasion mechanisms, including PD-L1 upregulation, T-cell exhaustion, and antigen-presenting cell heterogeneity (Zanotta et al., 2024).

### 7.7 Synergy with immune checkpoint inhibitors

Combination strategies using hTERT vaccination and immunotherapies targeting immune system checkpoints (ICIs) show potential in overcoming immunosuppression of the gynecologic tumor microenvironment. hTERT vaccines boost tumor-specific T-cell epitopes while PD-1/PD-L1 and CTLA-4 blocking suppresses effector function, improving infiltration and promoting lasting immunological responses (Fung and Kong, 2017; Xie et al., 2024). Mechanistically, vaccination-induced IFN gamma signaling enhances antigen presentation, and ICIs inhibit adaptive immune resistance mediated by PD-L1 induction (Herrmann, 2015). This combination makes gynecologic tumors, which are immunologically “cold,” more inflamed and provides an environment with high levels of T cells to support sustained tumor control (Butterfield and Najjar, 2024).

### 7.8 Approaches for synthetic lethality

Synthetic lethality approaches exploit telomere dysfunction-induced DNA damage dependencies in telomerase-inhibited tumors. Telomerase inhibition causes telomere dysfunction; in dividing cells, the resulting telomere dysfunction-induced foci (TIFs) engage the DNA damage response (DDR), particularly ATM/ATR-p53 signaling (Setton et al., 2021), which may trigger senescence or apoptosis. Importantly, unlike other genomic loci, telomere damage is largely unrepaired and persistent, meaning that DDR activation at telomeres predominantly drives cell death rather than repair-mediated survival (Fumagalli et al., 2012). By combining suppression of telomerase catalytic activity (*via* hTERT inhibition) with blockade of PARP or ATR/CHK1 signaling pathways, the DNA damage repair capacity of telomerase-inhibited tumour cells is overwhelmed, leading to replication stress and inducing chromosomal instability and apoptosis (Gu et al., 2024).

This approach is highly effective in ovarian cancers harboring pathogenic BRCA1/2 mutations, as defective homologous recombination in these cancers increases susceptibility to PARP inhibition (Gu et al., 2024). ATR and CHK1 inhibitors further overcome checkpoint control, which forces cells with critically short telomeres into lethal mitosis (Fernandez et al., 2025). Preclinical models show synergistic increases in gamma-H2A.X foci, telomere dysfunction-induced foci, and loss of clonogenic survival (i.e., inability of treated cells to form colonies, indicating permanent loss of proliferative capacity) with monotherapies (Tang et al., 2024).

### 7.9 Targeting ALT pathways

A subset of gynecological tumors are dependent on the ALT mechanisms for their telomere maintenance and thus requires an alternative approach to their treatment (Alnafakh et al., 2019). ALT-positive cancers have recombination-dependent telomere elongation, significant telomere heterogeneity, ALT-associated PML bodies (APBs), and increased replication stress (Amorim et al., 2016). Targeting ALT vulnerabilities, especially with ATR, FANCM, and CHK1 inhibition, causes selective lethality by interfering with telomere recombination and checkpoint control (Amorim et al., 2016; Berei et al., 2020). These approaches provide precision-based options for tumors resistant to telomerase inhibition (MacKenzie Jr et al., 2021).

#### 7.9.1 Resistance to telomerase-directed therapies

Resistance to telomerase-directed therapies represents a central barrier to clinical translation, arising through several adaptive mechanisms. Three principal pathways have been identified. First, activation of alternative lengthening of telomeres (ALT): upon sustained telomerase inhibition, a subset of tumour cells can switch to a homologous recombination–based telomere maintenance mechanism, thereby bypassing the intended therapeutic effect and preserving replicative potential (Hu et al., 2016). This ALT escape is associated with ATRX loss and represents a form of adaptive resistance that may emerge *de novo* under selection pressure rather than pre-existing as a fixed subpopulation (Napier et al., 2015). Second, shelterin remodelling: alterations in TRF1, TRF2, or POT1 can modify telomere capping efficiency and DDR signalling in ways that decouple telomere length from dysfunction, allowing cells with short telomeres to evade apoptosis or senescence (Hu et al., 2016). Mutations in POT1 have been reported in endometrial and ovarian cancers and may confer resistance by reducing the DDR signal at telomeres (Kim, W. T. et al., 2021). Third, tumour microenvironment (TME) adaptation: the TME can buffer telomere stress responses in gynaecological tumour cells through disease-specific mechanisms. In endometrial cancer, the oestrogen-rich stromal environment sustains telomerase *via* ERα-mediated hTERT transcription, potentially attenuating telomere-driven apoptotic signalling (Misiti et al., 2000; Kim et al., 1994). PTEN-loss-driven PI3K/AKT hyperactivation—a hallmark of endometrioid EC—additionally phosphorylates and stabilises hTERT independently of transcriptional upregulation (Lu and Broaddus, 2020). Whether PI3K/AKT activation in ovarian cancer peritoneal metastases promotes telomerase upregulation or ALT escape has not been demonstrated in disease-specific models and is a biologically plausible hypothesis requiring investigation (Apostolou and Fostira, 2013). In cervical cancer, persistent telomeric DNA damage signalling activates SASP-mediated inflammation, while HPV E7 disrupts IRF3-mediated innate immune sensing, preventing cGAS-STING recognition of cytosolic telomeric DNA fragments generated during chromosomal instability (Gabet et al., 2008). Cancer-associated fibroblasts may buffer telomere stress through HGF and IL-6 secretion, activating STAT3 and PI3K/AKT in tumour cells; however, direct gynaecological-specific evidence linking CAF paracrine signalling to telomere stress buffering remains limited and is identified as requiring empirical investigation. Where disease-specific evidence is insufficient, this is stated explicitly to distinguish established mechanisms from plausible hypotheses. Addressing TME-driven resistance requires combination strategies alongside patient stratification based on ALT status, shelterin mutation profiling, oestrogen receptor status, and TME composition.

#### 7.9.2 Plant-derived and lifestyle modulators of telomere biology

**FIGURE 5:**
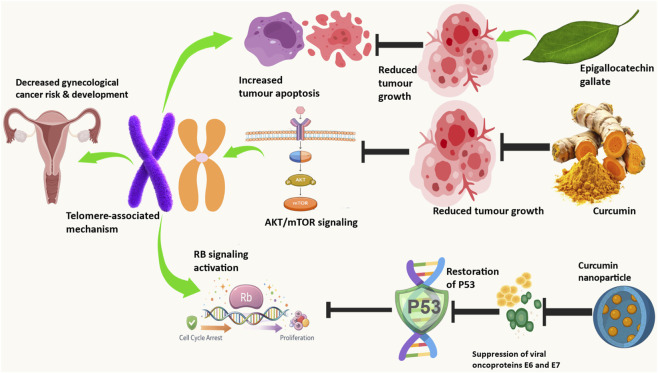
Impact of plant-derived interventions on gynecological cancer *via* telomere-associated mechanisms. This figure summarises preclinical and observational evidence on the telomere-associated effects of plant-derived compounds and lifestyle factors in gynaecological cancer models. Each row represents a distinct intervention, the cancer type or model studied, the primary biological outcome observed, and the putative telomere-associated mechanism reported. Direct telomerase inhibition has been demonstrated for EGCG in cervical cancer cell lines, while curcumin exerts indirect effects through suppression of HPV E6/E7 oncogenes that transcriptionally activate hTERT. Human observational data are limited to systemic leukocyte telomere length associations and do not reflect tumour-specific telomere biology. The strength of evidence is primarily preclinical and mechanistic inferences remain to be confirmed in clinical settings. *Figure was created using canva, Biorender and MS Powerpoint tools.

Mounting preclinical evidence demonstrates the modulatory impact of certain plant-derived compounds *via* telomere-associated mechanisms (Figure 5). Exposure of the green tea polyphenol epigallocatechin gallate (EGCG) in cervical cancer cell lines led to growth inhibition and apoptosis along with direct decrease in telomerase activity, which is a form of telomere maintenance that is necessary to maintain immortalization of cells (Yokoyama et al., 2004). Similarly, exposure to curcumin in HPV-positive cervical cancer cells inhibited the viral E6 and E7 oncogenes and restored p53 and Rb signaling; since HPV E6 is a known transcriptional activator of hTERT, this treatment suggested secondary telomerase inhibition, although telomere length was not measured (Vahedian Sadeghi et al., 2022). The available evidence based on human models are observational longer leukocyte telomere length was linked to healthier dietary and lifestyle patterns in a large cohort of women, implying slower systemic telomere erosion; however, no results regarding gynecological cancer incidence or tumor-specific telomere length were investigated (Cassidy et al., 2010).

Taken together, the existing data suggest that direct telomere or telomerase regulation in gynecological cancer is, first of all, supported only by preclinical cervical cancer models, and human data do not demonstrate tumor-specific telomere changes.

#### 7.9.3 Lifestyle and environmental factors

Systemic telomere dynamics and cancer risk are affected by dietary habits, physical exercise, stress management, metabolic health, and environmental exposures. Diets high in polyphenols and omega-3 fatty acids, caloric restriction, structured exercise, and stress alleviation reduce oxidative stress and inflammation, thereby maintaining telomeres, primarily at the systemic level (Baliou et al., 2024; Deng et al., 2016). Human studies evidence is mostly the reflection of telomere in the peripheral blood cells instead of tumor tissue, which shows that they have a role to play in risk modulation, treatment tolerance, and survival instead of direct tumor elimination. Telomere attrition is accelerated by obesity, metabolic syndrome, aging, oxidative stress, chronic inflammation, and environmental toxins that also subjugate hormone-sensitive carcinogenesis, especially endometrial cancer (Micallef Fava, 2022). Addressing these factors can have a beneficial effect on telomere-mediated genomic instability and supplement oncologic interventions (Table 1).

**TABLE 1:** Plant-derived and lifestyle modulators of telomere-associated mechanisms in gynecological cancers.

Exposure	Cancer type/Model	Key observed outcome	Telomere-associated mechanism reported	Evidence type	References
Epigallocatechin gallate	Cervical cancer cell lines	Growth inhibition and apoptosis	Direct telomerase activity reduction, impairing telomere maintenance	Preclinical (*in vitro*)	Yokoyama et al. (2004)
Curcumin (including nanoparticle formulation)	HPV-positive cervical cancer (HeLa) cells	Suppression of E6/E7 oncogenes; restoration of p53 and Rb	Indirect telomerase inhibition *via* E6 suppression (hTERT transcriptional downregulation implied); telomere length not measured	Preclinical (*in vitro*)	Vahedian Sadeghi et al. (2022)
Curcumin	Uterine leiomyosarcoma cells	Tumor growth suppression *via* AKT–mTOR inhibition	Telomerase involvement not directly assessed	Preclinical (*in vitro*)	Wong et al. (2014)
Diet and lifestyle factors (plant-rich diet, healthy behaviors)	Women (systemic leukocytes)	Longer leukocyte telomere length	Systemic telomere length preservation; no tumor-specific telomere analysis	Observational (human cohort)	Cassidy et al. (2010)

## 8 Conclusion and future perspectives

Gynaecological malignancies, such as malignancies of the ovaries, endometrium, cervix, vulva and vagina, are an essential and rising health burden for females worldwide. Despite the development of new strategies in prevention, molecular classification of the disease, and targeted treatment, clinical outcomes remain unsatisfactory for many patients, especially in the fields of ovarian cancer and the treatment of aggressive or recurrent endometrial disease. Genomic instability is at the forefront of telomere dysfunction and accounts for progression and therapeutic resistance of these malignancies.

A unifying conceptual model of telomere dysfunction across gynaecological cancers can be articulated around four shared mechanistic stages, each with subtype-specific departures. First, progressive telomere shortening driven by the end-replication problem and oxidative stress is common to all subtypes, but the rate and tissue context differ: rapidly cycling cervical epithelium and estrogen-stimulated endometrium are particularly vulnerable. Second, telomere crisis, characterised by BFB cycles, ATM/ATR activation, and genomic catastrophe, is the common tumorigenic inflection point, though its timing and intensity vary with checkpoint integrity. Third, telomere maintenance escape, predominantly *via* TERT reactivation (cervical, endometrial, most ovarian) or *via* ALT (clear cell and endometrioid ovarian, less commonly endometrial), determines the immortality pathway and dictates therapeutic sensitivity. Fourth, post-escape genomic evolution, shaped by ongoing shelterin disruption, TERRA dysregulation, and non-canonical TERT functions, produces the subtype-specific chromosomal landscapes that characterise each cancer. Mapping where each gynaecological cancer subtype sits within this framework clarifies both what is shared, the crisis-escape-evolution sequence, and what is cancer-specific, the molecular effectors at each stage, advancing the manuscript beyond descriptive cataloguing toward a genuinely explanatory framework.

Telomeres play a crucial role in the chromosomal integrity of gynaecological tissues, which undergo frequent cycles of proliferation, differentiation and hormonal regulation. Progressive telomere shortening, disorganisation of shelterin elements and aberrant activation of telomere maintenance pathways - most notably telomerase reactivation and, in a select subset of tumors, also alternative lengthening of telomere - contribute to the development of chromosomal instability, clonal evolution (whereby breakage-fusion-bridge cycles generate structural chromosomal variants enabling selection of increasingly malignant clones), and malignant transformation of various gynaecological cancers. Importantly, telomere biology interacts with female reproductive physiology, and therefore carcinogenesis is interconnected with processes such as ovarian ageing, endometrial remodelling, hormones, and fertility preservation.

Accumulating evidence suggests that the dynamics of telomere length, telomerase activity, and the DNA damage response are clinically relevant as biomarkers for cancer risk assessment, prognostic indicators, and indicators of therapeutic response. In addition, new therapeutic methods targeting telomere biology, such as telomerase inhibitors, telomerase-based immunotherapies, synthetic lethality approaches, and alternative lengthening of telomeres approaches, are promising candidates for precision oncology. Complementary strategies involving bioactive plant compounds and lifestyle interventions that target oxidative stress and inflammation may have further implications for telomere stability and cancer predisposition. In tumours that use the ALT pathway to maintain telomeres, the targeted disruption of telomere lengthening through recombination by inhibiting ATR, FANCM, and CHK1 provides a precise treatment strategy, as mentioned in the therapeutic strategies section above.

This review positions telomere dysfunction not merely as a factor in progressive malignant transformation, but also as a marker of mechanistic function and an amenable vulnerability of gynaecological cancers. There is enormous potential for the application of telomere biology in prevention, diagnostic tools, and the optimization of therapeutic outcomes across this broad spectrum of malignancies.

Although considerable progress has been made in understanding the role of telomere biology in gynaecological malignancies, numerous gaps remain that need to be bridged to enable clinical exploitation of this knowledge. Future research needs to move beyond descriptive relationships toward more integrative, far more mechanistically grounded, clinically stratified research within the framework of hormonal and reproductive factors in female tissues that are gender-specific.

A significant goal of interest is the development of a strong, tumor-specific telomere biomarker that goes beyond the blood bulk telomere length or hTERT expression only. Future studies should incorporate multi-parametric analyses, including telomere length heterogeneity, telomerase activation, shelterin, and DNA damage response signaling, with separate compartments within a tumor. Longitudinal sampling across diseases and treatment courses will be significant for defining telomere dynamics as predictors of therapeutic response, recurrence, and survival in ovarian, endometrial, and cervical cancers.

Given the critical influence of estrogen and progesterone on telomerase regulation, future studies should focus on cell-type- and hormone-specific, hormone-contextualised investigations of telomere maintenance. Single-cell and spatial transcriptomics, alongside telomere profiling, provide powerful tools to help resolve the intricate influence of hormonal signalling on telomere stability across a variety of epithelial, stromal, immune, and stem-like cell populations. Such approaches may be of particular relevance to hormonally responsive malignancies, whereby cyclical exposure to sex hormones, as well as receptor status, may potentially characterise telomere vulnerability and transformation into an oncogenic condition. Though intervention strategies targeting telomere-associated mechanisms are evolving to plant-derived compounds and lifestyle factors, evidence supporting direct modulation of tumor-specific dynamics by these interventions remains largely preclinical or indirect, with human studies primarily reflecting systemic leukocyte telomere length rather than tumor biology.

An unexplored, though very relevant, area of future research lies at the nexus of fertility preservation, cancer treatment, and telomere biology. Fertility-sparing therapies, particularly in endometrial cancer, are a unique and special clinical example to understand the impact of long-term hormonal modulation on telomere dynamics, genomic stability and long-term oncologic outcome. Prospective studies of telomere maintenance, combined with quantitative telomere and telomerase assessment, could provide insight into the potential role of telomere maintenance in explaining the correlation between fertility preservation strategies and cancer control.

While pharmacologic inhibition of telomerase still poses challenges due to toxic side effects and drug resistance, future studies should not only focus on pharmacologic inhibition but also examine adjunctive non-pharmacologic strategies that indirectly regulate telomere integrity, such as reducing oxidative stress, modulating mitochondrial biogenesis and metabolic dysfunction, and preventing inflammation. Notably, such interventions should be explored in the context of carefully designed clinical or translational investigations, with a clear demarcation between the systemic effects of telomere interventions and tumor-specific outcomes. This strategy may primarily pertain to long-term survivorship care and cancer prevention, rather than direct tumor destruction.

Lastly, further development of telomere-based strategies in gynaecological oncology would require conceptual models that integrate a multi-faceted view of reproductive ageing, hormonal control, and cancer susceptibility. Future studies would do better to adopt a longitudinal, life-course perspective and consider that telomere dynamics both influence and are influenced by reproductive lifespan, endocrine changes, and oncogenic stress. Such integrative frameworks may help achieve personalized risk stratification and inform prevention strategies based on the unique biological trajectories in female patients.
